# Supplementary material for: Vitamin B12 Status Upon Short-Term Intervention with a Vegan Diet—A Randomized Controlled Trial in Healthy Participants
Source: Nutrients. 2019 Nov 18;11(11):2815. doi: 10.3390/nu11112815 (PMC6893687; doi:10.3390/nu11112815)
Supplement: Supplementary file 1 [file nutrients-11-02815-s001.zip › Appendix.pdf]

UNIVERSITÄTSKLINIKUM FREIBURG
Institut für Umweltmedizin und Krankenhaushygiene
Department of Environmental Health Sciences
Breisacher Straße 115b, 79106 Freiburg

UNIVERSITÄTS KLINIKUM FREIBURG

Institut für Umweltmedizin und Krankenhaushygiene
Kommissarischer Leiter: Prof. Dr. Georg A. Häcker

Uni-Zentrum Naturheilkunde
Leiter: Prof. Dr. Roman Huber

Breisacher Str. 115 b, 79106 Freiburg
Tel 0761 270-82010
Fax 0761 270-83230
E-Mail: roman.huber@uniklinik-freiburg.de
www.uni-zentrum-naturheilkunde.de

Einfluss veganer Ernährung auf immunologische Parameter im Zusammenhang mit rheumatoider Arthritis bei Gesunden

Schulungsunterlagen für die vegane Kost

Sehr geehrte Probandinnen und Probanden,

hiermit erhalten Sie Ihre Schulungsunterlagen für die vegane Ernährung inklusive einiger Rezeptvorschläge für die kommenden vier Wochen.
Wir möchten Sie bitten, sich möglichst genau an unsere Vorgaben bei dieser Ernährungsweise zu halten und hoffen, dass wir Sie dabei mit den folgenden Hinweisen unterstützen können.
Sie erhalten von uns Gutscheine für ein werktägliches kostenloses Mittagsessen im Casino des Universitätsklinikums Freiburg.
Um Ihnen bei der Auswahl veganer Restaurants und Cafés in Freiburg zu helfen, haben wir bei einzelnen Lokalen eine Ermäßigung für die Zeit während der Studie verhandeln können. Sie erhalten dafür eine separate Bescheinigung, die Sie jeweils vor Ort als StudienteilnehmerIn kennzeichnet.
Wir bitten Sie, während der Studie

- keine Veränderungen Ihrer bisherigen Sportgewohnheiten vorzunehmen.
- an keinen anderen Studien oder Blutspenden teilzunehmen.
- keine Nahrungsergänzungsmittel einzunehmen.

Bitte tragen Sie alle verzehrten Lebensmittel in den ausgehändigten Ernährungs-protokollen ein und protokollieren Sie täglich nach der Morgentoilette Ihr Gewicht auf dem dafür vorgesehenen Bogen. Bei Rückfragen bezüglich der Ernährung melden Sie sich bitte bei:

Cand. med. Manuel Hettich
E-Mail: manuel.hettich@uniklinik-freiburg.de, Tel.: 01515/2255375

Was ist eine vegane Ernährung?

Eine vegane Ernährung definiert sich über die Beschränkung auf ausschließlich pflanzliche Lebensmittel.
Dies bedeutet, dass keinerlei Lebensmittel tierischen Ursprungs wie z. B. Fleisch, Wurst, Milch und Milchprodukte (Käse, Frischkäse, Quark, Joghurt, Butter, Sahne), Eier, Fisch und Honig konsumiert werden.

Wichtig: Gewichtsveränderungen

Pflanzliche Lebensmittel sind im Vergleich zu den meisten Lebensmitteln tierischen Ursprungs eher energiearm. Daher erhöht sich bei einer Betonung auf Obst und Gemüse im Vergleich zu ihren bisherigen Ernährungsgewohnheiten die Menge an Lebensmitteln und die jeweilige Portionsgröße, die Sie für den gleichen Kalorienbedarf konsumieren müssen. Vor allem am Anfang lohnt es sich deswegen, bewusst größere Mengen von den empfohlenen Lebensmitteln zu essen als bisher, um einem möglichen Gewichtsverlust vorzubeugen.
Falls sich Ihr Gewicht während der Studiendauer verändern sollte, geben Sie uns bitte Bescheid und versuchen Sie bei einem Gewichtsverlust mehr Nüsse, Öle und Vollkornprodukte zu konsumieren. Bei einem Gewichtsanstieg konzentrieren Sie sich bitte mehr auf energieärmere Lebensmittel wie Salate und Gemüse.

Vegane Ernährung im Alltag

- täglich reichlich Obst und Gemüse (frisch oder auch aus der Tiefkühlabteilung)
- mehrfach wöchentlich oder auch täglich frischen Salat
- täglich Hülsenfrüchte wie Linsen, Erbsen, Bohnen, Tofuprodukte (frisch gekocht, aus dem Glas, aus dem Tiefkühler)
- mehrfach wöchentlich oder auch täglich eine Portion Nüsse
- Leinsaat und Leinöl sind eine gute Quelle für Omega-3-Fettsäuren

Praxistipps:

- Kochen Sie eine größere Portion von Bohnen, Linsen oder Kartoffeln und verwenden Sie sie auch in der nächsten Mahlzeit oder frieren Sie sie zum Teil für später ein. Hierfür geeignet sind auch Reis, Hirse, Couscous oder Bulgur.
- Hülsenfrüchte sowie Reis und Hirse vor dem Kochen gut abspülen.
- Benutzen Sie hochwertige pflanzliche Öle zum Kochen.
- Versuchen Sie, verschiedene Tofuprodukte (marinierter Tofu, Tofuwürstchen, Aufschnitt) anstatt bisheriger Fleischkomponenten zu verwenden.
- Dünsten Sie Gemüse, verwenden Sie nur wenig Kochwasser um auch die wasserlöslichen Vitamine im Gemüse zu erhalten.
- Sojajoghurt, -pudding oder -quark in verschiedenen Geschmacksrichtungen können als ein einfacher Nachtisch dienen.
- Seien Sie neugierig und probieren Sie neue Rezepte oder Lebensmittel, die Sie noch nie zuvor gegessen haben!
- Es empfiehlt sich immer einen Packung Studentenfutter oder einen veganen Müsliriegel in der Tasche zu haben, falls Sie unerwartet Hunger bekommen.

Vegane Gerichte sind wahrscheinlich schon zuvor unbemerkt in ihrem Alltag vorgekommen oder Gerichte Ihres Alltags lassen sich problemlos veganisieren. Beispiele sind:

- Nudeln mit Tomatensoße
- Curry-Linsen-Reis
- Bulgur-/Couscoussalat
- Linsensuppe
- Gemüsecremesuppe (Sahne einfach durch Nussmus oder pflanzliche Sahne ersetzen)
- Burritos, Wraps oder Tacos mit Bohnen, Reis, Salat und Gemüse
- Chili sin carne mit Bohnen
- Burger oder Kebab mit gewürztem Veggie-Patty bzw. gebratenen Seitan-Streifen, Zwiebel, Pilzen und Paprika
- Milchreis mit pflanzlicher Milchalternative
- Kartoffelsalat mit Essig-Öl-Dressing
- Pestoschnecken (einfach Heteteig mit veganem Pesto bestreichen)
- Smoothies oder Säfte

Sie können auch von Beginn an selbst kreativ ans Werk gehen, indem Sie bei Ihrer Kochplanung die Teller-Methode anwenden:

- Füllen Sie die Hälfte Ihres Tellers mit nicht-stärkehaltigem Gemüse
- Füllen Sie ein Viertel Ihres Tellers mit einem Vollkornprodukt, Reis oder einem stärkehaltigen Gemüse wie Kartoffeln oder Mais
- Füllen Sie ein Viertel Ihres Tellers mit einer pflanzenbasierten Proteinquelle wie Hülsenfrüchte oder einem Tofuprodukt
- Fügen Sie eine Frucht, einen Salat und 1 Portion Nüsse hinzu
- Benutzen Sie verschiedene Gewürze und Kräuter, um den Geschmack zu variieren

Vegan im Supermarkt

Beispielweise können Sie in Ihren Einkaufswagen folgende Produkte packen:

- Reichlich Obst und Gemüse, auch als Tiefkühlvariante
- Kartoffeln, Zwiebeln, Knoblauch
- Nudeln, Reis, Hirse, Couscous, Bulgur, Buchweizen, Haferflocken
- Linsen, Bohnen, Erbsen, Tofuprodukte, Kichererbsen
- Nüsse, Trockenfrüchte, Saaten (Leinsaat, Sesam)
- Hefeflocken (statt Parmesan oder als Topping)
- Pflanzliche „Milch", Sojajoghurt, Agavendicksaft
- Margarine oder pflanzliche „Butter" (Alsan)
- Tomatenmark sowie Senf, Ketchup, vegane Mayonnaise
- Aufstriche (süß mit Schokolade oder Spekulatiuscreme oder herzhaft)
- Wenn es mal schnell gehen muss: Fertige Tomatensoße, fertiger Pizza- oder Blätterteig, Pommes, Gemüsestäbchen aus der Tiefkühltruhe
- Beispiele für den Süßhunger:
  Manner-Waffeln, Zartbitterschokolade (verschiedene Sorten), Marzipan-schokolade (Rittersport), Oreo-Kekse (Original), PastaFrutta (Haribo)

Rezeptvorschläge

Bananen-Müsli

Zutaten:

- 75 - 100g Haferflocken oder anderes Basismüsli
- 1 Banane, in Scheiben geschnitten
- 3 EL Apfelmus
- 2 TL Leinsamen
- ½ TL Zimt
- 1 EL Nüsse
- Soja-, Reis-, Hafer-, Kokosnuss-, oder Mandelmilch
- Falls vorhanden: frische Erdbeeren, Himbeeren oder Heidelbeeren

Vermischen Sie die Haferflocken mit den Leinsamen, dem Zimt und den Nüssen. Fügen Sie die Banane und das Apfelmus hinzu, sowie nach Belieben eine pflanzliche Milch-Alternative. Frische Beeren als Topping können dieses einfache Frühstück abrunden. Durch unterschiedliche Beeren oder Nüsse können täglich neue Variationen entstehen.

Porridge/Oatmeal

Zutaten

- 60g Haferflocken
- Wasser (oder Milchalternative)
- frisches Obst (nach Bedarf)
- 1 bis 2 EL Nussmus
- eine Prise Salz
- Je nach Geschmack: Zucker oder Zimt
- Nach Geschmack: Apfelmus, Sojajoghurt

Haferflocken in einem Topf kurz anrösten und dann mit Wasser (oder Milchalternative) ablöschen. Es sollte so viel Flüssigkeit hinzugegeben werden, dass das Wasser etwa zwei Fingerbreit über den Haferflocken steht. Aufkochen, dann bei niedriger Hitze weiterkochen lassen. Eine Prise Salz hinzugeben. Nussmus hinzugeben. Obst hinzugeben (wenn eine Erwärmung gewünscht ist). Wenn das Wasser verkocht ist, Porridge servieren, mit Obst vermischen und nach Geschmack mit Zimt, Zucker, Apfelmus, Sojajoghurt verfeinern.

Schokoladen-Chia-Pudding

Zutaten für 4 Portionen (über Nacht):

- 1 Tasse Chiasamen
- 1 ½ Tassen Soja-, Reis-, Hafer-, Kokosnuss-, oder Mandelmilch
- ¼ Tasse Kakaopulver
- 4 TL Ahornsirup

Vermischen Sie alle Zutaten in einer Schale und fügen Sie nach Belieben mehr Ahornsirup hinzu. Bedecken Sie die Schale im Kühlschrank für mindestens 3

Stunden, am besten über die Nacht. Servieren Sie den Pudding mit Ihren Lieblingsfrüchten, Nüssen oder Kakaobohnenbrüchstücken (Kakaonibs).

Guacamole

Zutaten für 4 - 6 Portionen:
- 4 reife Avocados
- 1 große Tomate
- ½ rote Zwiebel, fein geschnitten
- ½ Tasse Koriander, fein gewürfelt
- Falls gewünscht: 1 TL frische Jalapeños, gewürfelt
- Saft von 1 Zitrone
- Salz

Schneiden Sie die Avocados in Hälften und benutzen Sie einen Löffel, um das Avocadofleisch in einer Schale zu sammeln. Fügen Sie die restlichen Zutaten hinzu und vermischen Sie mit einer Gabel alles kräftig miteinander bis zur gewünschten Konsistenz. Abschließend können Sie nach Belieben Salz oder mehr Zitronensaft beigeben.
Tipp: Guacamole bzw. geöffnete Avocados nicht lange offen stehen lassen, sondern bis zum Verzehr luftdicht direkt abdecken. Ein Avocadokern auf der Guacamole kann bei der Lagerung helfen, einer schnellen Bräunung der Oberfläche vorzubeugen.

Hummus (benötigt einen Mixer)

Zutaten für 4 Portionen:
- 800g oder zwei Dosen gekochter Kichererbsen
- Saft von einer Kichererbsendose bzw. die gleiche Menge an Wasser
- 4 TL Tahin (Sesammus)
- 2 Knoblauchzehen
- Saft von 1 Zitrone
- 2 TL Olivenöl
- 1 TL Kreuzkümmel
- 1 Messerspitze Paprikagewürz
- Salz

Kippen Sie die Flüssigkeit einer Kichererbsendose ab und fangen Sie den Saft der anderen Dose auf. Waschen Sie die Kichererbsen gut ab. Mixen Sie alle Zutaten außer dem Saft der einen Kichererbsendose langsam und fügen Sie den restlichen Saft nach und nach hinzu, bis der Hummus cremig wird – möglicherweise benötigen Sie die gesamte Menge. Mixen Sie den Humus weiter, bis die gewünschte Konsistenz erreicht ist. Abschließend können Sie mehr Zitronensaft, Knoblauch, Kreuzkümmel und Salz zum Abschmecken hinzufügen. Verzieren Sie den Hummus in einer Schale mit etwas Paprikagewürz und ein paar Tropfen Öl.

Süßkartoffel-Wedges

Zutaten für 2 - 3 Portionen:
- 4 große Süßkartoffeln
- 1 TL geräucherte Paprika

- 1 TL Knoblauchgranulat
- Salz nach Belieben
- 1 TL Ahornsirup

Den Ofen auf 200°C vorheizen und die Kartoffeln waschen, die Schale muss nicht entfernt werden (idealerweise handelt es sich um Bio-Produkte). Schneiden Sie die Süßkartoffeln in die Wedge-Form und platzieren Sie sie in einer Schale. Fügen Sie das Paprikagewürz, Knoblauchgranulat, Salz und den Ahornsirup hinzu und vermischen Sie alles gut miteinander. Verteilen Sie die Wedges auf einem mit Backpapier ausgelegten Backblech und backen Sie sie für ca. 40 Minuten.
Dazu: Mayonnaise (ohne Ei, frei erhältlich) oder Ketchup.

Kichererbsen-Sandwich

Zutaten für 2 Portionen:

- Toastscheiben oder Brot Ihrer Wahl
- Für den Belag: Salat, Tomate, Avocado
- Für die Mischung:
  - 1 Dose gekochte Kichererbsen
  - 1 EL Tasse Sellerie
  - ½ rote Zwiebel, fein gewürfelt
  - 2 EL vegane Mayonnaise, gestampfte Avocado oder Hummus
  - Saft von ½ Zitrone
  - ½ TL Knoblauchpulver oder gehackter Knoblauch
  - 2 TL frischer Dill, gewürfelt (optional)
  - Salz, Pfeffer

Gießen Sie den Saft der Kichererbsen ab und zerstampfen Sie die Kichererbsen bis zur gewünschten Konsistenz in einer Schale. Vermischen Sie die Kichererbsen mit den restlichen Zutaten und fügen Sie nach Belieben Salz und Pfeffer hinzu. Nun können Sie aus der Mischung mit dem Brot und dem restlichen Belag Ihr Sandwich zusammenstellen.

Tomatensalat

Zutaten:

- Tomaten
- Knoblauch
- Zwiebeln
- Salz und Pfeffer
- ein EL Senf
- zwei EL Olivenöl
- Balsamicoessig nach Bedarf
- 1 TL Gemüsebrühe
- 1 EL Agavendicksaft

Essig mit Agavendicksaft, Senf, Gemüsebrühe, Öl und Gewürzen verrühren. Zwiebeln und Knoblauch von Schale befreien und klein schneiden. Zu Essigmischung hinzugeben. Tofu in Würfelschneiden. Zu Essigmischung hinzugeben. Wenn möglich 24 Stunden im Kühlschrank ziehen lassen. Tomaten

waschen und klein schneiden, mit Tofu und Essigmischung verschmischen, ggf. nachwürzen.

Tofugulasch

Zutaten für 4 Personen:

- 3 Kartoffeln
- ¼ Sellerieknolle
- 4 EL Olivenöl
- 3 EL Tomatenmark
- 1 Dose gestückelte Tomaten (400g)
- 80 ml Rotwein
- 250 ml Wasser
- 3 EL Shoyu-Sojasauce
- evtl. 1 kleine rote Chilischote
- 400g Tofu
- Meersalz
- 2 Zweige Rosmarin
- ½ TL Oreganoblättchen
- 1 TL Paprikapulver edelsüß

Karotten und Sellerie schälen, klein würfeln und im heißen Olivenöl leicht braun anbraten. Das Tomatenmark zugeben und anbraten. Mit Dosentomaten, Rotwein, Wasser und der Sojasauce ablöschen.
Die Chilischote halbieren, entkernen und fein hacken. Den Tofu gleichmäßig in 1 ½ cm große Würfel schneiden und vorsichtig unterrühren. Alles 20 Minuten leise köcheln lassen und regelmäßig vollständig, aber sanft umrühren. Mit Salz abschmecken, die Gewürze dazugeben und weitere 10 Minuten garen. Die Rosmarinzweige vor dem Servieren entfernen.
Beilagen: Polenta oder Reis und ein Salat

Linseneintopf
(für 2-3 Portionen)

Für zwei Portionen:

- 3 mittelgroße Kartoffeln
- 2 Karotten
- 80g Tellerlinsen
- 1 Paprika
- 400-500ml Gemüsebrühe
- 3 EL Passierte Tomaten
- 1 Schuss Essig (bevorzugt Balsamico)
- Kräuter der Provence
- Salz & Pfeffer
- 1 EL Rapsöl

Kartoffeln und Karotten klein schneiden und in Gemüsebrühe kochen. Wenn das Wasser kocht, Linsen hinzugeben und eine halbe Stunde bei mittlerer Temperatur einkochen. Wenn ein Großteil der Flüssigkeit verdampft ist, gut würzen und Tomaten, Essig und Öl hinzufügen. Dann weiter einkochen.
Kurz vor Ende Paprika hinzugeben.

Panierter Tempeh

Zutaten für 4 Personen:

- 400g Tempeh (fermentierte Sojabohnen)
- 60g Ingwer
- 2 EL Sambal Oelek
- 6 EL Reismehl
- 2 EL Maisstärke
- 10 EL kaltes Wasser
- Meersalz
- 6 EL Sesamöl nativ

Den Tempeh in 8mm dicke Scheiben schneiden. Den Ingwer schälen und klein reiben, den Saft in eine Schüssel pressen und mit Sambal Oelek, Reismehl, Maisstärke, Wasser und Salz zu einem dünnen Teig rühren. Den Tempeh kurz darin wenden und in einer beschichteten Pfanne im heißen Sesamöl von beiden Seiten 5 Minuten goldbraun anbraten.
Beilagen: Curry- oder Kokosreis, gebratene Auberginen mit Tomaten und ein Salat

„Käse“-Nudeln

Zutaten für 3 – 4 Portionen:

- ½ Tasse Cashewnüsse, für 1 Std. eingeweicht
- 2 mittelgroße (Süß-)Kartoffeln
- 1 TL Tamari / Sojasauce
- 2 Knoblauchzehen
- Saft von 1 Zitrone
- 2 gehäufte TL Hefeflocken
- 1 TL Kurkuma
- 1 Messerspitze Paprikagewürz
- Makkaroni-Nudeln

Lassen Sie die Cashewnüsse für eine Stunde in warmen Wasser einweichen. Waschen Sie die Kartoffeln und schneiden Sie sie in Würfel, ggf. entfernen Sie die Rinde. Kochen Sie die Kartoffeln bis zu einer weichen Konsistenz und die Makkaroni-Nudeln nach der Packungsanleitung und gießen Sie anschließend das Kochwasser ab. Mixen Sie die Cashewnüsse mit den Hefeflocken, etwas Wasser, den Kartoffeln, dem Kurkuma, Knoblauch und dem Zitronensaft bis zur gewünschten Konsistenz. Sie können dabei, falls erforderlich, noch weiteres warmes Wasser hinzufügen. Vermischen Sie die Sauce gut mit den Makkaroni und bestreuen Sie die Nudeln vor dem Servieren mit ein paar Hefeflocken und etwas Paprikagewürz.

Kartoffel-Spinat-Auflauf mit Cashewkruste

(für eine kleine Auflaufform)

- 4-5 Kartoffeln
- 300g Spinat (TK)
- 50g Cashewkerne
- 1 Zwiebel

- 1 Knoblauchzehe
- etwas Öl
- 3 EL Zitronensaft
- Kräuter (nach Geschmack)
- Salz & Pfeffer

Vorarbeit:
Etwa 3-4 Stunden vor der Zubereitung: Spinat aus dem Tiefkühlfach nehmen und auftauen lassen.
Etwa 1 Stunde vor der Zubereitung: Cashewkerne mit Wasser einweichen.
Etwa eine Viertelstunde vor der Zubereitung: Kartoffeln waschen und mit Schale in etwas Wasser kochen

Hauptarbeit:
Ofen auf 180°C vorheizen.
Zwiebel klein schneiden und in etwas Öl anbraten. Cashews abgießen und zusammen mit den angebratenen Zwiebeln in den Mixer. Dazu Salz, Pfeffer, Knoblauch, Kräuter und Zitronensaft. Kartoffeln mit Schale in Scheiben schneiden und in die Auflaufform. Spinat darauf schichten und die Masse aus dem Mixer darüber geben. Dann für ungefähr 30min in den Ofen.

Kirsch-Käsekuchen

Zutaten für 1 Springform von 26cm Durchmesser:

- Mürbeteig
    - 100g pflanzliche Margarine (z.B. Alsan)
    - 80g Rohrohrzucker
    - 250g (Dinkel-)Mehl (Type 630)
    - 120g gemahlene Mandeln
- Füllung
    - 400g Seidentofu
    - 2 Päckchen Vanillepudding-Pulver
    - 1 gestrichener TL Agar-Agar
    - Abrieb von 1 Bio-Zitrone
    - 200ml Sojasahne (z.B. Cresoy)
    - 400g frische oder 300g tiefgekühlte Kirschen
    - 50g Rohrohrzucker
    - etwas Sahnesteif

Mürbeteig: Margarine, Zucker und Mehl mit dem Knethaken zu einem festen bröseligen Teig vermengen. Die Springform einfetten und den Teig einfüllen. Zuerst den Rand 3 cm hoch locker andrücken und dann die restlichen Krümel noch einmal auf dem Boden gleichmäßig verteilen und leicht andrücken. Die Mandeln auf dem Boden ausbreiten.
Füllung: Den Seidentofu mit Puddingpulver und Agar-Agar fein pürieren. Die Zitronenschale unterrühren. Die Sojasahne mit etwas Sahnesteif für ca. 10 Min. steif schlagen und vorsichtig unter den Tofu ziehen.
Fertigstellung: Den Backofen auf 170°C vorheizen. Die Füllung in die Kuchenform gießen und die entsteinten, eventuell gefrorenen Kirschen darauf verteilen. Mit dem Zucker bestreuen und den Kuchen im Ofen gut 40 Minuten golden backen. Den

Kuchen weitere 10 Minuten im geschlossenen ausgeschaltenen Ofen ruhen lassen, damit die Sojasahne nicht zusammenfällt.
Mit einer Teigkarte den Tortenrand lösen, den Springformrand öffnen und das Konditorblech unter den Kuchen schieben. Mindestens eine Stunde auskühlen lassen.

Schoko-Tahin-Kuchen

Zutaten für 1 Springform von 26cm Durchmesser:

- Teig
    - 320g (Dinkel-)Mehl (Type 630)
    - 120g Rohrohrzucker
    - 140g pflanzliche Margarine (z.B. Alsan)
    - 250ml Reisdrink Natur
    - 125g weißes Tahin (Sesampaste)
    - 1 Päckchen Backpulver
    - 50g Kakaopulver
    - 30ml Reisdrink Natur
- Glasur
    - 200g Zartbitterkuvertüre
    - 100ml Sojamilch Vanille
    - 1 EL weißes Tahin (Sesampaste)
    - 2 EL Wasser

Teig: Den Backofen auf 170°C vorheizen und Mehl, Zucker, Margarine, 250ml Reisdrink, Tahin und Backpulver zu einem schaumigen Teig verquirlen. Die Hälfte davon in die gefettete Springform füllen. Die andere Hälfte mit dem Kakaopulver und restlichem Reisdrink aufschlagen. Auf dem hellen Teig gleichmäßig verteilen und mit einer Gabel locker durch den Teig fahren, dabei von unten nach oben durchziehen, sodass eine schöne Marmorierung entsteht. Den Kuchen im Ofen 40 Minuten backen.
Den Kuchen aus dem Ofen nehmen und den Springformrand lösen. Etwas auskühlen lassen und den Kuchen mit einer Konditorplatte von dem Springformboden nehmen.
Glasur: Die Kuvertüre mit Sojamilch in eine Schüssel geben und über einem heißen Wasserbad schmelzen. Das Tahin mit dem Wasser in einer separaten Schüssel zu einer dickflüssigen Sauce verrühren.
Den ausgekühlten Kuchen mit der Glasur überziehen und einige Tropfen Tahin darauf geben. Ist die Schokolade fest geworden, kann der Kuchen aufgeschnitten werden.

UNIVERSITÄTSKLINIKUM FREIBURG
Institut für Umweltmedizin und Krankenhaushygiene
Department of Environmental Health Sciences
Breisacher Straße 115b, 79106 Freiburg

Institut für Umweltmedizin und Krankenhaushygiene
Kommissarischer Leiter: Prof. Dr. Georg A. Häcker

Uni-Zentrum Naturheilkunde
Leiter: Prof. Dr. Roman Huber

Breisacher Str. 115 b, 79106 Freiburg
Tel 0761 270-82010
Fax 0761 270-83230
E-Mail: roman.huber@uniklinik-freiburg.de
www.uni-zentrum-naturheilkunde.de

Einfluss veganer Ernährung auf immunologische Parameter im Zusammenhang mit rheumatoider Arthritis bei Gesunden

Schulungsunterlagen für die fleischreiche Ernährung

Sehr geehrte Probandinnen und Probanden,

hiermit erhalten Sie Ihre Schulungsunterlagen für die fleischreiche Ernährung inklusive einiger Rezeptvorschläge für die kommenden vier Wochen.
Wir möchten Sie bitten, sich möglichst genau an unsere Vorgaben bei dieser Ernährungsweise zu halten und hoffen, dass wir Sie dabei mit den folgenden Hinweisen unterstützen können.
Sie erhalten von uns Gutscheine für ein werktägliches kostenloses Mittagsessen im Casino des Universitätsklinikums Freiburg.
Um Ihnen bei der Auswahl von Fleischgerichten in Restaurants und Metzgereien in Freiburg zu helfen, haben wir bei einzelnen Betrieben eine Ermäßigung für die Zeit während der Studie verhandeln können. Sie erhalten dafür eine separate Bescheinigung, die Sie jeweils vor Ort als StudienteilnehmerIn kennzeichnet.
Wir bitten Sie, während der Studie

- keine Veränderungen Ihrer bisherigen Sportgewohnheiten vorzunehmen.
- an keinen anderen Studien oder Blutspenden teilzunehmen.
- keine Nahrungsergänzungsmittel einzunehmen.

Bitte tragen Sie alle verzehrten Lebensmittel in den ausgehändigten Ernährungsprotokollen ein und protokollieren Sie täglich nach der Morgentoilette Ihr Gewicht auf dem dafür vorgesehenen Bogen. Bei Rückfragen bezüglich der Ernährung melden Sie sich bitte bei:

Cand. med. Manuel Hettich
E-Mail: manuel.hettich@uniklinik-freiburg.de, Tel.: 01515/2255375

Was ist eine fleischreiche Ernährung?

Durch die DGE (Deutsche Gesellschaft für Ernährung e. V.) wird für eine aufgewogene Mischkost eine Verzehrempfehlung für Fisch für zwei- bis dreimal pro Woche angegeben, zusätzlich sollten pro Woche nicht mehr als 300 bis 600 Gramm Wurstwaren und Fleisch verzehrt werden. Bei höheren Verzehrmengen liegt eine fleischreiche Ernährung vor.

Wichtig: Gewichtsveränderungen

Pflanzliche Lebensmittel sind im Vergleich zu den meisten Lebensmitteln tierischen Ursprungs eher energiearm. Daher verringert sich bei einer Betonung auf tierische Lebensmittel im Vergleich zu ihren bisherigen Ernährungsgewohnheiten die Menge an Lebensmitteln und die jeweilige Portionsgröße, die Sie für den gleichen Kalorien-bedarf konsumieren müssen. Vor allem am Anfang lohnt es sich deswegen, bewusst kleinere Mengen von den empfohlenen Lebensmitteln zu essen als bisher, um einer möglichen Gewichtszunahme vorzubeugen.

Falls sich Ihr Gewicht während der Studiendauer verändern sollte, geben Sie uns bitte Bescheid und versuchen Sie bei einem Gewichtsanstieg die jeweiligen Portions-größen zu verringern. Bei einem Gewichtsverlust erhöhen Sie bitte die einzelnen Portionsgrößen, wobei Sie in jedem Fall die tägliche bzw. wöchentliche Frequenz der vorgegebenen Lebensmittelarten einhalten sollten.

Fleischreiche Ernährung im Alltag

- Täglich mindestens eine Fleisch- oder Fischkomponente (mindestens 150g)
- Morgens und abends eine Milchkomponente (z. B. Milch, Joghurt, Butter oder Käse)
- Wöchentlich mindestens drei verarbeitete oder ganze Eier
- Zu jeder Hauptmahlzeit Fleisch, Fisch, Eier und/oder ein Milchprodukt
- Legen Sie in den nächsten vier Wochen Ihren Fokus auf das Essen von tierischen Lebensmitteln und reduzieren Sie dafür den Verzehr von Salat, Gemüse und Obst.
- Verzichten Sie auf Produkte, die den Titel „vegan“ tragen.
- Achten Sie darauf, dass Sie, wenn sie unterwegs Hunger bekommen, beim Bäcker oder Imbiss ein Gericht mit Fleisch oder zumindest einem tierischen Lebensmittel wählen (Brötchen mit Wurst oder Fisch, Seele mit Speck und Käse, Käsestange, süße Teilchen mit Quark).

Praxistipps:

- Essen Sie als Nachtisch einen Joghurt oder einen Pudding statt Obst.
- Achten Sie auf eine tierische Komponente bei süßem Frühstück (beispiels-weise Butter, Frischkäse oder Quark unter die Marmelade).
- Hartgekochte Eier (nach etwa 8 bis 10 Minuten) lassen sich mehrere Tage im Kühlschrank aufheben und sind auch einer guter Snack für unterwegs.
- Fleischreste lassen sich problemlos einfrieren.
- Fleisch, das nicht rasch verbraucht wird, lässt sich ebenso problemlos einfrieren.
- Bei Fisch immer auf Kühlung achten.

- Kaufen Sie Fleisch- und Fischprodukte frisch und verbrauchen Sie sie rasch!
- Achten Sie darauf die Arbeitsbereiche Ihrer Küche gründlich zu reinigen, wenn Sie mit Eiern, Fisch oder Fleisch gearbeitet haben.
- Viele Sorten Fleisch lassen sich auch roh verzehren (beispielweise Hackfleisch als Mettbrötchen).
- Sie können Säfte, süße Getränke oder Smoothies auch durch Buttermilch, Joghurt- oder Milchmixgetränke ersetzen.
- Viele Gerichte lassen sich mit Fleischkomponente verfeinern (beispielsweise Speck im Kartoffelsalat oder auf Flammkuchen).
- Aus Fleischresten können Sie eine Basis für Soßen einkochen.
- Fleisch nur kurz anbraten und dann bei niedrigeren Temperaturen durchgaren (wahlweise auch im Backofen).
- Fleisch ruhen lassen.
- Fleischsaft auffangen für Saucen oder Suppen (kann auch im Kühlschrank aufgehoben werden).
- Verwenden Sie statt pflanzlicher Fette Schmalz, Butter oder Ghee.
- Seien Sie neugierig und probieren Sie neue Rezepte oder tierische Lebensmittel, die Sie noch nie zuvor gegessen haben!

Ihre alltägliche Ernährung lässt sich leicht mit Produkten tierischen Ursprungs anreichern. Beispiele sind:

- Nudeln mit Tomatensoße und Käse
- Eintöpfe mit Sahne verfeinern oder mit Würstchen oder Speck anreichern
- Toppings für Salat: Fetakäse, Schinken- oder Speckwürfel
- Kartoffelsalat mit Speck verfeinert
- Bratkartoffeln mit Speck oder Schinkenwürfeln
- Milch oder Sahne im Kaffee
- Obst nicht pur, sondern in Joghurt oder Quark
- Datteln oder auch Bohnen im Speckmantel
- Pizza in verschiedenen Varianten (mit Salami, Schinken, Meeresfrüchten)
- Gefüllte Blätterteigtaschen (beispielweise mit Hackfleisch oder Salami)
- Pfannkuchen mit Sahne und heißen Kirschen oder als herzhafte Variante mit Schinken und Käse

„Reich an tierischen Produkten" im Supermarkt

Beispielweise können Sie in Ihren Einkaufswagen folgende Produkte packen:

- Verschiedene Wurstvarianten wie Lyoner, Fleischwurst oder auch italienische Salami (frisch oder abgepackt)
- Steak, Schnitzel, Gulaschfleisch, Kotelett, Geschnetzeltes (Frischetheke)
- Markknochen zum Einkochen von Suppe und zur Herstellung von Soßen
- Geflügelfleisch (Brathähnchen, Putenbrust, Wurstvarianten)
- Eingemachte Wurstspezialitäten wie Mettwurst oder Leberwurst
- Hackfleisch (Rind-, Schwein- oder gemischte Variante)
- Speck in verschiedenen Varianten
- Schmalz (mit Speck, Röstzwiebeln, pur)
- Schinken in verschiedenen Varianten (z. B. Walnuss- oder Trüffelschinken)
- Verschiedene Pasteten

- Wurst- bzw. Fleischsalat (z. B. von der Frischetheke)
- Geräuchter Lachs, Forellenfilet, Garnelen
- Fisch aus der Tiefkühltheke (Garnelen, Lachsfilet)
- Sämtliche Käsesorten wie beispielweise Gouda, Emmentaler, Parmesan, Brie, Münster, Fetakäse (abgepackt oder von der Frischetheke)
- Frischkäse (Ziege, Käse, Kuh) natur oder mit Kräutern oder Honig
- Frische Milch
- Buttermilch, Kefir, Ayran, Trinkjoghurts, Milchmixgetränke
- Joghurt, Quark (auch als herzhafte Kräuterquarkvariante), Skyr
- Butter, Ghee zum Anbraten
- Sahne, Schmand, Mascarpone
- Süße Aufstriche wie Nuss-Nougat-Creme oder Schokoladencreme, Honig
- Mayonnaise
- Eier
- Es empfiehlt sich frische Fleisch- und Milchprodukte sowie Eier auf dem örtlichen Markt (häufig bessere Qualität und Frische) zu beziehen.
- Auch Fleischwaren sind beim örtlichen Metzger häufig in besserer und frischerer Qualität zu erhalten.
- Wenn Sie die Möglichkeit haben, beziehen Sie auch Wildprodukte vom örtlichen Jäger (häufig auch auf Märkten zu finden).
- Wenn es mal schnell gehen muss: „Klassisches Fastfood“ wie Doseneintopf, Fischstäbchen, Tiefkühlpizza, Mozzarellasticks
- Fast alle Sorten Süßwaren enthalten tierische Produkte (andernfalls sind die Produkte als „vegan“ gekennzeichnet)

Rezeptvorschläge

Lasagne

Zutaten:

- 400g Hackfleisch
- 1 Zwiebel
- 100ml Sahne
- 1 Packung Rahmsoßenpulver
- 2 EL Mehl
- 150g geriebener Käse (z.B. Gouda-Käse)
- Lasagneplatten

Das Hackfleisch mit der gehackten Zwiebel anbraten, mit dem Mehl bestäuben und mit Wasser ablöschen. Das Rahmsoßenpulver hinzugeben und zusammen aufkochen lassen, zwischendurch regelmäßig umrühren, bis sich eine gleichmäßige Soße ergibt. Die Sahne hinzugeben und mit Salz und Pfeffer abschmecken. In einer Backform mit der Hackfleischsoße beginnend, abwechselnd Lasagneplatten und Hackfleischsoße schichten. Zum Schluss mit geriebenem Käse bestreuen und für 45 Min. bei 180°C im vorgeheizten Ofen backen.

Strammer Max

Zutaten:

- zwei Scheiben frisches Brot
- zwei Scheiben Schinken
- zwei Scheiben Käse
- zwei Eier
- Butter
- Essiggurken
- Senf
- Salz und Pfeffer
- Fett zum Braten
- Frische Kräuter

Brotscheiben auf einen Teller legen und mit Butter bestreichen. Mit einer Prise Salz und Pfeffer bestreuen. Brot mit Schinken und Käse belegen. Fett in einer Pfanne erhitzen, sobald eine ausreichende Hitze erreicht ist beide Eier aufschlagen und anbraten (nach kurzen Anbraten von etwa 1-2 Minuten Hitze reduzieren und etwa 5-6 Minuten durchgaren lassen). Essiggurken der Länge nach aufschneiden und auf die Schinkenbrote legen. Gebratene Eier auf vorbereitete Brote geben, mit Senf bestreichen und mit Kräutern garnieren.

Panierte Schnitzel

Zutaten:

- zwei Schnitzel (egal welcher Tierart)
- Paniermehl
- ein Ei
- Fett zum Anbraten

Paniermehl in einen Suppenteller füllen, ein Ei von der Schale befreien und in einem Suppenteller mit einer Gabel verquirlen. Beide Teller bereitstellen. Schnitzel waschen, mit einem Küchentuch trocknen, sehnige Stücke und Fettstücke entfernen. Flach auf das Küchenbrett legen und mit einem Fleischklopfer platt klopfen. Pfanne mit Fett erhitzen. Schnitzel zunächst in Ei eintauchen und gut benetzen, dann in Paniermehl wälzen. Anschließend kurz (bis eine leichte Bräunung eintritt) auf beiden Seiten braten und dann die Hitze reduzieren. Bei niedriger Hitze für etwa 10 Minuten durchgaren lassen (optional auch im warmen Backofen).

Garnelen in exotischer Sahnesoße

Zutaten:

- Garnelen (aus der Tiefkühlabteilung)
- Zwiebeln und Knoblauch
- Sojasoße
- Fleischbrühe
- eine Karotte
- eine Handvoll Tiefkühlspinat
- Sahne
- Salz und Pfeffer
- Currygewürzmischung

Zwiebeln und Knoblauch von der Schale befreien und klein schneiden. Karotte klein schneiden, Tiefkühl-Spinat bereitstellen. Fett in einer Pfanne erhitzen. Zwiebeln und Knoblauch anbraten. Sobald beides eine leichte Bräunung hat mit Sojasoße ablöschen und kurz karamellisieren lassen. Anschließend mit etwas Brühe aufgießen und bei reduzierter Hitze köcheln lassen. Karottenstücke und Spinat hinzufügen. Salz, Pfeffer und Curry hinzugeben. Je nach gewünschter Schärfe reichlich Curry hinzufügen. Mit Sahne abschmecken. Garnelen waschen und anschließend hinzugeben. Köcheln lassen, bis Garnelen gar sind.

Soßengrundbasis

Zutaten:

- Fleischreste (z. B. nach Schnitzelzubereitung)
- Salz und Pfeffer
- Muskatnuss
- eine Prise Zucker
- einen Schluck Rotwein
- Sahne

Zwiebel und Knoblauch von der Schale befreien und klein schneiden. Fleischreste gemeinsam mit Zwiebeln und Knoblauch in reichlich Fett anbraten (es sollte nichts

anbrennen) bis Fleischsaft austritt. Eine Prise Zucker hinzugeben und kurz karamellisieren lassen. Dann mit Rotwein ablöschen. Hitze reduzieren und köcheln lassen. Mit Salz, Pfeffer und Muskatnuss abschmecken. Etwas Sahne hinzugeben und bei niedriger Hitze köcheln lassen bis gewünschte Sämigkeit erreicht ist.

Mehlschwitze als Soßenbasis

Zutaten:
- Butter
- Mehl (Typ 405)
- Milch
- Salz und Pfeffer
- Muskatnuss

Butter in einem kleinen Topf verflüssigen. Sobald die Butter flüssig ist, unter ständigem Rühren langsam Mehl hinzufügen. Es sollte eine sämige Masse entstehen. Hitze reduzieren. Etwas Milch hinzufügen, Soße unter regelmäßigem Rühren einkochen lassen. Mit Salz, Pfeffer und Muskaltnuss abschmecken.

Für eine Sahnesoße Sahne hinzufügen.
Für eine Käsesoße Stücke von kräftigem Käse wie älterer Gouda oder Bergkäse hinzufügen und langsam unter Rühren verflüssigen.

Rindergulasch

Zutaten:
- 500 – 750g Ochsenfleisch
- Zum Schmoren: 40-50g Butter
- 2-3 Zwiebeln, Paprika
- evtl. ½ Knoblauchzehe
- ½ l kochende Brühe, Salz
- nach Belieben: 2-3 geschälte Tomaten, 1 Paprikaschote, Thymian, etwas Zitronenschale
- zum Binden: 1-2 EL Mehl
- zum Verbessern: etwas Tomatenmark, 4 EL Rahm, etwas Zitronensaft, etwas Wein

Fleisch kurz waschen, in ca. 2cm breite Würfel schneiden, Zwiebeln in Scheiben, Tomaten vierteln und Paprikaschote von Kernen und Rippen befreien und in feine Streifen schneiden.
Fleisch ungesalzen in heißem Fett bei guter Hitze rasch von allen Seiten kräftig in offenem Bratentopf anbräunen (größere Fleischmengen portionsweise anbräunen um starkes Saftziehen zu vermeiden. Wenn der Saft eingesogen ist und das Fleisch schön gebräunt ist, die Zwiebeln hinzugeben, etwas bräunen lassen, mit Paprika bestauben, leicht anrösten. Nach Belieben ein wenig Knoblauch beifügen, mit nur wenig kochender Flüssigkeit aufgießen, salzen, vorbereitete Tomaten, Paprikastreifen und Thymian beifügen, zugedeckt bei mäßiger Hitze gar schmoren unter allmählicher Zugabe geringer Flüssigkeitsmenge je nach Bedarf. Kurz vor Ende

der Garzeit mit Mehlteiglein binden, Verbesserungszutaten beifügen, noch etwas 10 Min. durchziehen lassen. Zitronenschale nur kurz in Soße belassen, dann entfernen, abschmecken. Man kann auch angebräuntes Fleisch und Zwiebeln mit trockenem Mehl bestäuben, etwas bräunen lassen, dann aufgießen und fertigstellen wie oben. Diese Art erfordert mehr Sorgfalt beim Schmoren; Binden durch Mehlteiglein entfällt dann. Garzeit etwa ½ Stunden. Im Dampfdrucktopf verkürzt sich die Kochzeit auf etwa 15 Min.
Beilagen: Salzkartoffeln, Kartoffelbrei, Knödel aus gekochtem Kartoffelteig, Semmelknödel, Spätzle oder Teigwaren; Salate nach Belieben.

Schinken mit Rührei

Zutaten:

- Pro Person: 1-2 frische Eier, 1-2 EL Milch, Salz, Schnittlauch
- evtl. zum Strecken pro Ei: ½ TL Mehl und 1EL Milch
- 50g magerer Schinken, roh oder gekocht
- Zum Garen: 10g Butter

Eier mit der Milch, dem Salz und evtl. mit Streckzutaten mit Schneebesen gut verschlagen, fein geschnittenen Schinken und Schnittlauch untermischen. Die Butter in einer Pfanne schmelzen lassen, Eiermasse hinzugeben, etwas anziehen lassen, Masse bei mäßiger Hitze unter öfterem leichtem Zusammenschlagen andicken lassen, bis die Masse flockig, aber noch weich ist - sie zieht nachträglich noch etwas an.
Beilage: grüner Salat

Schweinemedaillons, Schweinesteaks gebraten

Zutaten:

- 4 (8) Scheiben ausgelöste Schweinslende (oder -filet)
- Zum Würzen: 1-2 TL Zitronensaft, 2 EL Öl, ¼ TL Curry, 1 Messerspitze Pfeffer
- 60g Butter, Salz

Steaks mit einem feuchten Tuch leicht abreiben, mit der Hand etwas flachklopfen. Die Würzzutaten gut mischen, Steaks auf beiden Seiten damit einstreichen, aufeinanderlegen, etwa ½ - 1 Stunde zugedeckt kaltstellen. Vorbereite Steaks in heißer Butter auf beiden Seiten in einer Pfanne goldbraun braten, Bratdauer je Seite 4-6 Minuten. Steaks sollen gar, innen aber noch saftig sein. Leicht salzen und anrichten.
Beilagen: Gedünstetes Gemüse, Salate, nach Belieben etwas Ketchup oder Remouladensoße, Weißbrot oder Pommes frites.

Geschnetzeltes Fleisch Schweizer Art

Zutaten:

- ½ kg Ochsenfleisch oder Kalbsfilet bzw. -lende
- Etwas Zitronensaft

- Zum Schmoren: 40-50g Fett (halb Öl, halb Butter), 1 Zwiebel, 1 EL Mehl, ¼ l kochende Brühe, Salz
- Zum Verbessern: 4-6 EL Rahm, etwas Wein nach Belieben

Sehr gut abgehangenes Fleisch mit einem feuchten Tuch abreiben, in dicke Scheiben schneiden, diese klopfen, mit Zitronensaft beidseitig marinieren und zugedeckt ½ - 1 Stunde kaltstellen. Dann mit einem scharfen Messer gegen die Faser in feine Scheibchen schneiden. Feingeschnittene Zwiebel in heißem Fett in einer Pfanne andünsten, Fleischscheibchen hinzugeben und sofort bei guter Hitze rasch anbräunen – sofort Mehl drüberstreuen und bei guter Hitze leicht anbräunen, mit wenig kochender Brühe aufgießen, salzen, zugedeckt garen, mit Rahm und etwas Wein abschmecken, sofort zu Tisch geben. Garzeit bis zu 30 Minuten. Sehr zartes Fleisch nur ein paarmal aufkochen lassen, zu langes Garen macht Fleisch trocken und faserig. Wenig zartes Fleisch ist dafür nicht geeignet.
Beilagen: Reis, Kartoffelbrei, Butterkartoffeln mit Petersiele, Spätzle oder Teigwaren; Gemüse und Salate nach Wahl.

Kartoffelsalat mit Fleischsalat

Zutaten für 4 Portionen:
- 6-8 mittelgroße Kartoffeln, festkochend
- 200g Fleisch-Salat
- ½ kl. Glas Salatcreme
- 4 Eier, hart gekocht
- 1-2 mittelgroße Zwiebeln
- 2 EL Senf, 1 EL Essig, 1 EL Mineralwasser
- Salz und Pfeffer
- Petersilie
- evtl. Knoblauchgranulat

Alle Zutaten klein schneiden und vermischen. Zwiebeln nicht zu fein hacken, auch die Eierstücke nicht zu klein schneiden. Falls gewünscht eine geringe Menge an Knoblauchgranulat hinzufügen, keinen frischen Knoblauch verwenden. Der Salat sollte eine (leichte) Gelbfärbung haben. Etwas Petersilie unterheben, mit dem Rest den Salat garnieren und mindestens 12 Std. ziehen lassen. Aufgrund der großen Kalorienmenge nur kleinere Portionen als Beilage verwenden.

Schinkennudeln

Zutaten:
- 250g Bandnudeln, Spaghetti oder Makkaroni mit reichlich Salzwasser
- 20-40g Butter
- 100 – 250g gek. Schinken
- Nach Belieben: 2-3 Eier, 2 EL Milch, Salz, 30g Reibkäse, Petersilie

Nudeln in reichlich kochendem Salzwasser kernig-weich kochen, abgießen und gut abtropfen lassen. Die Nudeln in einer Pfanne mit der heißen geschmolzenen Butter kurz anrösten lassen bis die Nudeln gut heiß sind. Den klein geschnittenen Schinken

untermengen, nach Belieben die mit Milch und Salz verquirlten Eier darüber geben und so lange rösten, bis die Eier angezogen haben. Zuletzt nach Belieben mit geriebenem Käse und Petersilie bestreut anrichten.
Beilagen: Tomatensoße, grüner Salat.

Wurstsalat

Zutaten für vier Portionen:

- 500g magere Fleischwurst
- 2-3 Zwiebeln
- 50 – 100g Emmentaler Käse
- Zur Marinade: 2-3 EL Essig, 2-3 EL Öl, Salz, etwas Senf, 1 Prise Pfeffer

Kalbfleischwurst, Schinkenwurst, Bierschinken, Lyonerwurst o.ä. häuten und in feine Scheiben, Streifen oder Würfel schneiden. Mit feingeschnittenen Zwiebelringen oder Zwiebelwürfeln und nach Belieben mit feingeschnittenen Käsescheiben oder Käsewürfelchen mischen. Die Salatmarinade aus den genannten vermischten Zutaten gut abschmecken, den Salat damit anmachen und kaltstellen. Nach Belieben beim Anrichten mit etwas Pfeffer bestreuen, mit Tomatenscheiben oder Eischeiben verzieren.

Tiramisu

Zutaten:

- 4 frische Eigelb
- 4 EL Zucker
- 500 g Mascarpone
- Zitronenschale
- 150 g Löffelbiskuits
- 200 ml kalter Espresso
- 2 EL Kakaopulver (ungesüßt)

Eigelb und Zucker mit den Schneebesen des Handrührgerätes mind. 5 Minuten dickcremig schlagen. Mascarpone löffelweise unterrühren. Zitronenschale zufügen. Die Hälfte der Löffelbiskuits nebeneinander in eine flache Form legen. Die Hälfte des Espressos über die Biskuits träufeln. Die Hälfte der Mascarponecreme auf die Biskuits streichen. Übrige Kekse nebeneinander darauf verteilen und mit dem restlichen Espresso beträufeln. Übrige Mascarponecreme darauf streichen. Drei Stunden kalt Stellen und dann mit Kakao bestäuben.

Käsekuchen

Zutaten:

Teig:

- 125 g Zucker
- 125 g Butter
- 1 Vanillezucker

- 1 Ei
- 250 g Mehl + ein halbes Päckchen (ca. 2 Kaffeelöffel) Backpulver

Belag:

- 500 g Magerquark
- 500 g Quark mit 40 %
- 200 g Zucker
- 1 Vanillezucker
- 3 Eier
- 1 halber Becher süße Sahne
- 3 gehäufte EL Mondamin

Zusätzlich 1 Eigelb und etwas Sahne um die Torte zu überziehen.

Butter und Zucker schaumig schlagen, Vanillezucker und Ei dazu rühren, Mehl mit Backpulver dazu, in eine 28cm Form geben und kurz ruhen lassen. Quark, Zucker, Vanillezucker, Eigelb, Sahne und Mondamin verrühren, steifgeschlagenes Eiweiß unterheben, in die Form geben. Am Ende Kuchen mit dem in Sahne verrührten Eigelb überziehen.

Bei 200° C für 50 min - 60 min backen (für Umluft etwa 175°C), im Verlauf Temperatur ggf. etwas reduzieren (je nach Backofen kann der Kuchen sonst dunkle Flecken bekommen. Am Ende im Backofen auskühlen lassen.
