# Supplementary material for: Vitamin B12 Status Upon Short-Term Intervention with a Vegan Diet—A Randomized Controlled Trial in Healthy Participants
Source: Nutrients. 2019 Nov 18;11(11):2815. doi: 10.3390/nu11112815 (PMC6893687; doi:10.3390/nu11112815)
Supplement: Supplementary file 1 [file nutrients-11-02815-s001.zip › Appendix2.pdf]

Freiburger Ernährungsprotokoll

vom:

bis:

Anzahl der Tage:

Tragen Sie für jedes von Ihnen verzehrte Lebensmittel einen Strich in die vorgesehene Spalte ein. Sollt ein Lebensmittel nicht vorhanden sein, so markieren Sie ein ähnliches oder nutzen Sie die freien Zeilen und fügen es handschriftlich dazu.

Bitte beachten Sie die Portionsgröße und machen Sie ggr. mehrere Striche, z.B. für eine große Tasse Kaffee zwei Striche bei "Kaffee kleine Tasse", für 5 Plätzchen fünf Striche bei in der Zeile "Plätzchen Stücke". Beachten Sie, wenn möglich, Mengenangaben auf der Verpackung, ggf. bitte wiegen. Tragen Sie bei einer Mahlzeit alle Lebensmittel einzeln ein, z.B. erhält bei Bratwurst mit Brötchen, Pommes und Cola jedes der vier Lebensmittel einen Strich. Erfassen Sie alle Getränke und alles, was Sie zwischen den Mahlzeiten essen.

Führen sie das Protokoll am besten ständig mit und füllen sie es möglichst an Ort und Stelle aus.

Abkürzungen: Bech. = Becher / Port. = Portion / St.= Stück / EL= Eßlöffel / TL= Teelöffel / Sch.= Scheibe

Lebensmittel	Einheit	Anzahl	Σ
**Brot**			
Brötchen	St. 45 g		
Croissant	St. 50 g		
Graubrot	Sch. 45 g		
Hefezopf	Sch. 45 g		
Knäckebrot	Sch. 10 g		
Toastbrot	Sch. 20 g		
Vollkornbrötchen	St. 55 g		
Vollkornbrot	Sch. 50 g		
Weißbrot	Sch. 35 g		
Zwieback	Sch. 10 g		
**Brotbelag**			
Butter für 1 Scheibe Brot	TL 5 g		
Margarine für 1 Scheibe Brot	TL 5 g		
Margarine halbfett s. o.	TL 5 g		
Edelpilzkäse	Sch. 30 g		
Frischkäse	EL 30 g		
Schmelzkäse	Port. 30 g		
Schnittkäse 30%F.i.Tr.	Sch. 30 g		
Schnittkäse 50% F.i.Tr.	Sch. 30 g		
Weichkäse 45% F.i.Tr.	Sch. 30 g		
Weichkäse 60 % F.i.T.	Sch. 30 g		
Bierschinken	Sch. 25 g		
Corned Beef	Port. 25 g		
Fleischwurst	Sch. 20 g		
Fleischkäse (Aufschnitt)	Sch. 30 g		
Fleischsalat	Port. 50 g		
Leberwurst	Port. 30 g		
Mettwurst	Port. 30 g		
Teewurst	Port. 30 g		
Salami/Cervelatwurst	Sch. 20 g		
Schinken roh	Sch. 15 g		
Schinken gekocht	Sch. 30 g		
Speck	Port. 30 g		
Honig	EL 20 g		
Konfitüre	EL20 g		
Nuß-Nougat-Creme	EL 20 g		
Vegetabiler Brotaufstrich	Port. 30 g		
**Frühstücksallerlei**			
gekochtes Ei	St. 55 g		
Cornflakes	EL4 g		
Cornflakes gezuckert/geröstet	EL 6 g		
Haferflocken	EL 10 g		
Müsli	EL 15 g		
**Milch / Milchprodukte**			
Buttermilch	Glas 200 g		
Joghurt natur fettarm (1,5%F.)	Bech. 150 g		
Joghurt natur vollfett (3,5% F.)	Becher 150 g		
Joghurt mit Frucht fettarm (1,5%F.)	Bech. 150 g		
Joghurt mit Frucht vollfett (3,5% F.)	Bech. 150 g		
Milch fettarm (1,5% F.)	Gl 200 g		
Milch vollfett (3,5% F.)	Gl 200 g		
Kakao/Trinkschokolade	Gl 200 g		
Quark Magerstufe	EL 20 g		
Quark Halbfettstufe	EL20 g		
Sahne (30 % F.)	EL 10 g		
Kondensmilch (7,5% F.)	Port. 12 g		
**Obst**			
Brombeere, Erdbeere, Himbeere, Johannisbeere, Heidelbeere	Beerenobst Port. 125 g		
Weintraube	Port. 150 g		
Apfel, Birne, Quitte,...	Kernobst Port. 150 g		
Aprikose, Kirsche, Mirabelle, Pflaume, Pfirsich,...	Steinobst Port. 150 g		
Banane	St. 120 g		
Ananas, Kiwi, Mango, Maracuja,...	Südfrüchte Port. 150 g		
Grapefruit, Mandarine, Orange, Zitrone	Zitrusfrüchte Port. 150 g		
Rosinen, Trockenobst	Port. 50 g		
**Sonstiges**			
Cornichons, saure Gurken	St. 50 g		
Nüsse	Port. 100 g		
Oliven	Port. 100 g		
Erdnüsse gesalzen	Tasse 100 g		
Erdnußflips	Tasse 50 g		
Chips	Tasse 30 g		
Salzstangen	Port. 30 g		
**Suppen / Eintöpfe**			
als Vorsuppe			
Suppe klar	Port. 200 g		
Suppe gebunden	Port. 200 g		
Cremesuppe	Port. 200 g		
Gulaschsuppe	Port. 200 g		
Nudelsuppe m. Huhn	Port- 200 g		
als Hauptgericht			
Gemüsesuppe	Port. 400 g		
Kartoffelsuppe	Port. 400 g		
Linseneintopf	Port. 400 g		
**Fleisch / Fisch**			
Hackfleisch	Port. 100 g		
Kalbfleisch	Port. 230 g		
Rindfleisch	Port. 200 g		
Schweinefleisch	Port. 200 g		
Innereien	Port. 170 g		
Kotelett	Port. 170 g		
Schnitzel paniert	Port. 200 g		
Würstchen	Port 100 g		
Brathähnchen (1/2)	Port. 370 g		
Geflügel	Port. 170 g		
Fisch	Port. 175 g		
Fischfilet paniert	Port. 200 g		
Fischkonserve abgetr.	Port. 65 g		

Beilagen

Kartoffeln (Salzkart.)	Port. 200 g		
Pellkartoffeln	Port. 200 g		
Bratkartoffeln	Port. 200 g		
Kartoffelbrei	Port. 200 g		
Kartoffelknödel	St. 100 g		
Kartoffelpuffer	St. 75 g		
Kartoffelsalat	Port. 250 g		
Pommer Frites	Port. 200 g		
weißer Reis gekocht	Port. 180 g		
Natur-Reis gekocht	Port. 180 g		
Nudeln eifrei gekocht	Port. 180 g		
Vollkornnudeln gekocht	Port 180 g		
Semmelknödel	St. 100 g		
Schupfnudeln	Port. 400 g		
Spätzle, Eiernudeln gekocht	Port. 200 g		

Soßen und Fette

Joghurt-Salat-Soße	Port. 40 g		
Essig-Öl-Marinade	Port. 20 g		
Bechamelsoße	Port. 75 g		
Grundsoße	Port. 75 g		
Hackfleischsoße	Port. 100 g		
Jägersoße	Port. 75 g		
Käsesoße	Port. 75 g		
Grüne Soße, Kräuterquark	Port 200 g		
Tomatensoße	Port. 75 g		
Grillsauce	Port. 20 g		
Tomatenketchup	Port. 20 g		
Tomatenmark	TL 6 g		
Senf	TL 6 g		
Mayonnaise (80% F.)	EL 12 g		
Bratfett	EL 10 g		
Pflanzenöl	EL 10 g		

Gemüse / Salate

Blattsalat mit Dressing	Port. 60 g		
Rohkostsalat mit Dressing	Port. 180 g		
Bleichsellerie, Mangold, Spinat	Blattgemüse Port. 200 g		
Grüne Bohnen	Port. 200 g		
Aubergine, Gurke, Paprika, Tomate, Zucchini	Frucht-gemüse Port. 200 g		
Gemüsemais	Port. 200 g		
Blumenkohl, Broccoli, Kohl (Rot-, Grün-, Weiß-), Kohlrabi, Rosenkohl, Wirsing	Kohlgemüse Port. 200 g		
Sauerkraut	Port.150 g		
Fenchel, Lauch, Spargel, Zwiebel	Sprossen-gemüse Port 200 g		
Möhre, Radieschen, Rettich, Rote Bete, Rüben, Sellerie, Schwarzwurzel	Wurzel- und Knollen-gemüse Port. 200 g		
Pilze	Port. 120 g		

Fertig- und Schnellgerichte

Nudelsalat	Port. 250 g		
Wurstsalat	Port. 250 g		
Griechischer Salat	Port. 300 g		
Italienischer Salat	Port. 300 g		
Brotwurst ohne Brötchen	St. 150 g		
Currywurst ohne Brötchen	St. 150 g		
Hamburger	St. 100 g		
Cheeseburger	St. 120 g		
Big Mac	St. 200 g		
Maultaschen / Ravioli	Port 200 g		
Pizza	St. 400 g		
Pfannkuchen	Port. 300 g		

Hülsenfrüchte

Bohnen	Port. 200 g		
Erbsen	Port. 200 g		
Linsen	Port. 200 g		

Dessert/Kuchen / Süßes

Pudding	Port.150 g		
Eiscreme	Kugel 50 g		
Obstkuchen	St. 120 g		
Cremetorte	St. 150 g		
Rührkuchen	St. 60 g		
Platzchen, Kekse	St. 10 g		
Schokolade	Riegel 20 g		
Praline	St. 10 g		
Bonbon, Hartkaramelle	St. 3 g		
Fruchtgummi	Port. 50 g		
Zucker	TL 5 g		

Getränke

Kaffee	kleine Tasse 150 g		
Tee	kleine Tasse 150 g		
Mineralwasser	Glas 200 g		
Limonade	Glas 200 g		
Colagetränke	Glas 200 g		
Obst- Fruchtsaft	Glas 200 g		
Obst- Fruchtnektar	Glas 200 g		
Bier alkohofrei	Glas 330 g		
Bier	Glas 330 g		
Weizenbier	Glas 500 g		
Weißwein	Glas 200 g		
Rotwein	Glas 200 g		
Sekt	Glas 100 g		
Likör	Glas 40 g		
Schnaps, Branntwein	Glas 20 g		

Hier haben Sie noch einmal die Möglichkeit Lebensmittel aufzuschreiben, die auf der Liste nicht aufgeführt sind und die Sie verzehrt haben!

Name:
Vorname:
Adresse:
Tel.Nr:/ Fax/Mobil

Ggf. E-Mail:
